# Preoperative inflammatory indicators predict postoperative SIRS after cardiac surgery with cardiopulmonary bypass in older patients

**DOI:** 10.1186/s12877-026-08075-5

**Published:** 2026-08-07

**Authors:** Xiaoqian Wang, Xue Yang, Yongqing Cheng, Xiaotian Chen

**Affiliations:** 1 Department of Clinical Nutrition, Nanjing Drum Tower Hospital, Affiliated Hospital of Medical School, Nanjing University, Nanjing, Jiangsu China; 2 Department of Cardiac Surgery, Nanjing Drum Tower Hospital, Affiliated Hospital of Medical School, Nanjing University, Nanjing, Jiangsu China

**Keywords:** Cardiovascular surgery, Older, Neutrophil‑lymphocyte ratio, Systemic immune‑inflammation index, Systemic inflammation response index, Systemic inflammatory response syndrome

## Abstract

**Background:**

Systemic inflammatory response syndrome (SIRS) is a significant postoperative complication in older patients leading to higher morbidity and mortality rates. This study investigates the predictive value of preoperative inflammation markers for postoperative SIRS in older adults undergoing cardiac surgery with cardiopulmonary bypass.

**Methods:**

This retrospective study screened 290 patients ≥ 65 years. Patients were categorized into two groups based on the development of postoperative SIRS. Inflammatory indices including neutrophil-to-lymphocyte ratio (NLR), systemic inflammatory index (SII) and systemic inflammation response index (SIRI) were calculated based on preoperative blood routine examination. The discriminatory power of each marker was evaluated with ROC curve analysis, and the odds ratio (OR) was used to examine the relationship between inflammatory indices and postoperative SIRS.

**Results:**

Ninety patients (31.0%) fulfilled SIRS criteria within 3 days after surgery. Patients with postoperative SIRS showed significant variations in NLR, SII, and SIRI compared to those without. ROC analysis showed that NLR ≥ 2.8, SII ≥ 1066, and SIRI ≥ 1.05 effectively predicted postoperative SIRS. Multivariate analysis identified NLR ≥ 2.8 (OR = 1.94, 95% CI: 1.06–3.77), SII ≥ 1066 (OR = 2.74, 95% CI 1.53–4.92), and SIRI ≥ 1.05 (OR = 2.46, 95% CI 1.30–4.67) as independent predictors for postoperative SIRS development. The predictive value of these inflammatory indices for postoperative SIRS was consistent across subgroups. Patients complicated by postoperative SIRS had longer ICU stays, extended mechanical ventilation duration, higher ICU readmission rates, and greater long-term all-cause mortality.

**Conclusion:**

We showed that NLR ≥ 2.8, SII ≥ 1066, and SIRI ≥ 1.05 can effectively predict postoperative SIRS in older cardiac surgery patients, helping clinicians identify high-risk individuals for early intervention.

**Supplementary Information:**

The online version contains supplementary material available at https://doi.org/10.1186/s12877-026-08075-5.

## Introduction

Despite decades of medical evolution, cardiac surgery with cardiopulmonary bypass (CPB) still provoke systemic inflammatory response syndrome (SIRS), which is a complex physiological reaction characterized by the release of different inflammatory mediators and the activation of the immune system [1, 2]. Relevant studies indicate that postoperative SIRS rates vary significantly, ranging from 9% to 29%, influenced by factors such as study design, hospital ward, patient demographics, and medical conditions [3, 4]. Studies have shown that SIRS following surgery is closely associated with an increased risk of postoperative morbidity and mortality [5].

CPB triggers a strong inflammatory response from blood interacting with synthetic materials, flow changes, ischemia-reperfusion, endotoxemia, and oxidative stress, which can activate pathways and cells leading to SIRS and potentially multiorgan dysfunction if not managed [6]. However, in older patients, the response becomes markedly dysregulated due to progressive immunosenescence and chronic low-grade inflammation, making it harder for them to effectively control the intensity and duration of post-surgery inflammation [7]. Though older patients have a lower incidence of postoperative SIRS than younger patients, SIRS in the elderly predicts severe consequences [8]. Such patients suffer significantly higher risks of all-cause death, stroke/transient ischemic attack, requirement for renal replacement therapy, major bleeding, and postoperative intra-aortic balloon pump implantation [9].

To reduce the risk of postoperative SIRS, preoperative assessment and postoperative monitoring are crucial. A panel of preoperative inflammatory biomarkers has been validated to facilitate the preoperative risk stratification of postoperative SIRS, which comprises the C-reactive protein/hyperlipoprotein ratio (CHR), platelet-to-lymphocyte ratio (PLR), and neutrophil-to-lymphocyte ratio (NLR) [10–12]. Regarding cardiovascular surgeries involving CPB, studies show that preoperative NLR shows moderate ability to predict postoperative infection [13]. Even though the relationship between inflammatory indicators and SIRS has been documented in various medical and surgical settings, it is rarely reported in older patients after cardiac surgery.

The early prediction of postoperative SIRS is essential for optimizing perioperative management, particularly in improving the prognosis of elderly patients. This study aimed to identify older patients at high risk for postoperative SIRS by utilizing routinely measured preoperative inflammatory biomarkers. These biomarkers can be readily integrated into clinical practice, thereby aiding anesthesiologists and clinicians in implementing early interventions to prevent the onset of SIRS.

## Methods and materials

### Patients

Consecutive patients over the age of 65 who underwent a cardiovascular operation with CPB between Jan 2020 to Dec 2020 in our hospital, were retrospectively included in this study. Patients were excluded for preoperative infection, chronic renal or liver dysfunction, aortic dissection, intraoperative or 72-hour postoperative death, confirmed postoperative infection within 3 days after surgery, or incomplete study data. Following the exclusion criteria, 290 patients remained in the study (Figure S1). The patients were subsequently split into two categories: the SIRS group and the non-SIRS group, based on their postoperative SIRS development. The study protocol was sanctioned by the Hospital Clinical Research Ethics Committee (no.2022-216-01). Informed consent and clinical trial registration requirements were waived by the committee.

### Data collection

Patient information was collected from the hospital’s registry system and patient records. Demographic features (including age, gender, and additional diseases), preoperative medications such as angiotensin-converting enzyme (ACE) inhibitors, Statins, Steroids, admission laboratory tests (including complete blood count and serum biochemical profiles), nutritional status assessed by the Controlling Nutritional Status (CONUT) score, operative details (intraoperative procedural durations, medications, mechanical circulatory support and transfusion), and postoperative data (clinical parameters of SIRS and in-hospital short-term outcomes) were all recorded. The CONUT score, NLR, systemic inflammatory index (SII) and systemic inflammation response index (SIRI) values were calculated according to the blood routine examination and biochemical results.

In addition, long-term outcomes post-discharge data were gathered prospectively through individual phone calls using a structured questionnaire (Appendix S1) at predetermined intervals.

### Study outcomes

The primary endpoint of the study was prevalence of SIRS. We evaluated the incidence of SIRS within the first three postoperative days. The clinical parameters assessed in all patients included: (1) body temperature below 36 °C or above 38 °C, (2) heart rate exceeding 90 beats per minute, (3) respiratory rate greater than 20 breaths per minute or arterial carbon dioxide tension (PaCO_2_) below 32 mmHg, and (4) white blood cell count less than 4 × 10^9^/L, greater than 12 × 10^9^/L, or with more than 10% immature bands [12]. The prevalence of SIRS was determined by the presence of at least two of the above mentioned clinical criteria. Postoperative secondary endpoints included ICU stay, hospital stay, mechanical ventilation duration, ICU readmission rate, in-hospital mortality, and long-term all-cause mortality.

### Statistics

We displayed the continuous variables as either a mean ± standard deviation (SD) or a median with an interquartile range. The analysis was performed using an unpaired Student’s t-test or Mann-Whitney U test because the data were not normally distributed. Frequencies and percentages were used to present the categorical variables, which were then analyzed with a proportions test. Receiver operating characteristic (ROC) curves were documented and area under the curve (AUC) values were computed to assess and compare the ability of preoperative inflammatory indicators to discriminate. The Youden index (J = [sensitivity + specificity] − 1) was employed to find the best predictive cutoffs for AUC value calculation. Univariable regression analysis was used to preliminarily analyze the risk factors for the occurrence of postoperative SIRS (Table S2). Statistically significant variables (*p* < 0.05) on univariable logistic regression analysis were included in the multivariable logistic regression to identify independent risk factors. Collinearity diagnostics confirmed the absence of multicollinearity among all variables incorporated into the multiple regression model. Odds ratio (OR) was calculated with a 95% confidence interval (CI). The test by Hosmer-Lemeshow suggested good calibration in the absence of significance. In addition, the clinical stratification analysis aimed to determine the predictive value of preoperative inflammatory indicators for postoperative SIRS, based on clinical factors like gender, age, body mass index (BMI), hypertension, and diabetes.

## Results

### Study population

A total of 290 patients were included in this analysis. The number of patients with SIRS occurrence within 72 h from surgery was 90 (31.0%). The SIRS and non-SIRS group did not differ significantly regarding gender (*p* = 0.930), age (*p* = 0.636), BMI (*p* = 0.598), NYHA functional class ( *p* = 0.259), comorbid conditions, intraoperative characteristics (Table 1), and the preoperative use of stains (Table S1). Patient groups also differed significantly with respect to surgical type, blood white blood cells, blood neutrophils and fasting plasma glucose (*P* < 0.05). Crucially, intergroup differences in NLR, SII and SIRI reached statistical significance, with all P values < 0.05.

**Table 1:** Patient characteristics

Variables	Non-SIRS ( *n* = 200)	SIRS ( *n* = 90)	*P*-value
Age, years	71 (68–74)	70 (68–74)	0.636
Male sex	90 (45.00%)	40 (44.44%)	0.930
BMI, kg/m^2^	23.69 ± 3.78	23.44 ± 3.70	0.598
NYHA functional class ≥ 3	92 (46.00%)	35 (38.89%)	0.259
Comorbid conditions			
COPD	1 (0.50%)	2 (2.22%)	0.203
Diabetes	22 (11.00%)	11 (12.22%)	0.762
Hypertension	98 (49.00%)	45 (50.00%)	0.875
Stroke	18 (9.00%)	7 (7.78%)	0.732
CONUT ≥ 2	121 (60.50%)	61 (67.78%)	0.236
Type of surgery			0.007
Valve	120 (60.00%)	40 (44.44%)	
Valve + CABG	27 (13.50%)	18 (20.00%)	
Aorta	6 (3.00%)	11 (12.22%)	
Other	47 (23.50%)	21 (23.33%)	
Intraoperative characteristics			
DOS (min)	253 (205–315)	260 (211–343)	0.140
CPB time (min)	130 (107–164)	138 (103–173)	0.117
ACC time (min)	96 (74–124)	100 (75–132)	0.223
Laboratory parameters			
WBC, ×10 ^9^/L	5.3 (4.5–6.7)	6.2 (5.2–9.1)	< 0.001
Neutrophils ×10 ^9^/L	3.1 (2.4–4.4)	4.1 (3.0-6.9)	< 0.001
Lymphocytes×10 ^9^/L	1.5 (1.2–1.8)	1.5 (1.1–1.9)	0.893
PLT×10 ^9^/L	158 (123–190)	164 (125–200)	0.365
Hb, g/L	128.01 ± 18.35	126.92 ± 19.05	0.643
TSB, mg/dL	0.68 (0.53–0.98)	0.73 (0.47–1.06)	0.141
SUA, umol/L	369 (312–459)	372 (304–455)	0.626
Cr, mg/dL	66 (57–78)	68 (54–77)	0.704
TG, mmol/L	0.98 (0.72–1.28)	1.04 (0.73–1.42)	0.340
HDL-C, mmol/L	1.16 (0.94–1.41)	1.12 (0.96–1.34)	0.712
LDL-C, mmol/L	2.30 ± 0.80	2.23 ± 0.79	0.520
Albumin, g/L	38.6 (36.5–40.2)	38.0 (36.6–40.4)	0.975
FPG, mmol/L	4.64 (4.31–5.16)	4.72 (4.30–5.68)	0.021
eGFR, mL/min/1.73 m^2^	93.30 ± 24.36	93.36 ± 26.15	0.984
NLR	2.1 (1.5–3.2)	3.0 (2.0-5.1)	< 0.001
SII	1155 (771–1623)	1476 (1085–2063)	< 0.001
SIRI	0.74 (0.51–1.32)	1.43 (0.73–2.79)	< 0.001

*BMI* body mass index, *NYHA* New York Heart Association, *COPD* chronic obstructive pulmonary disease, *CONUT* Controlling Nutritional Status, *CABG* coronary artery bypass grafting, *DOS* duration of surgery, *CPB* cardiopulmonary bypass, *ACC* aortic cross clamp, *WBC* white blood cells, *PLT* platelet count, *Hb* hemoglobin, *TSB* total serum bilirubin, *SUA* serum uric acid, *Cr* creatine, *TG* triglyceride, *HDL-C* high-density lipoprotein cholesterol, *LDL-C* low-density lipoprotein cholesterol, *FPG* Fasting plasma glucose, *eGFR* estimated glomerular filtration rate, *NLR* neutrophil-to-lymphocyte ratio, *SII* systemic inflammatory index, *SIRI* systemic inflammation response index

### Receiver Operator Characteristics (ROC) analysis

**Fig. 1:**
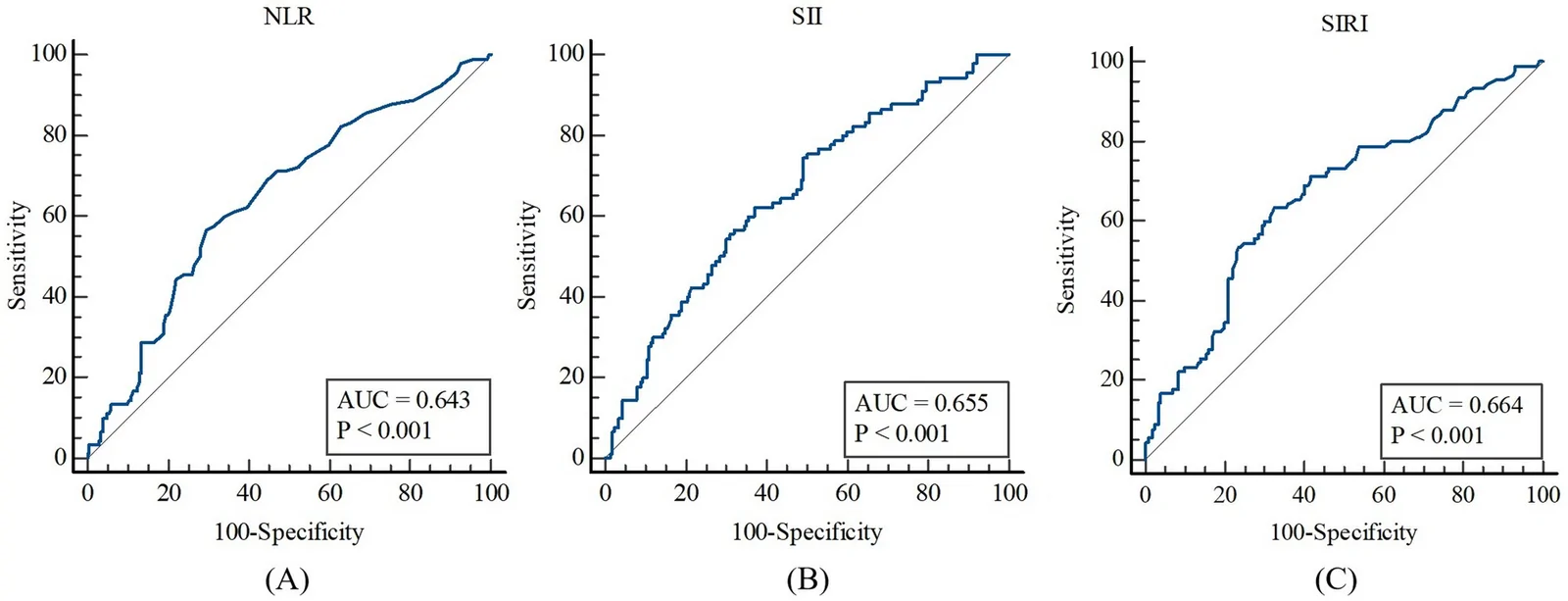
Receiver operator characteristics curves for NLR (**A**), SII (**B**), and SIRI (**C**). Abbreviations: NLR-neutrophil-to-lymphocyte ratio, SII-systemic inflammatory index, SIRI- systemic inflammation response index

Using MedCalc software, the ROC curve was created based on the predicted probability and actual instances of postoperative SIRS. The predictive abilities of the biomarkers for systemic inflammation were evaluated and compared using AUC values. ROC analysis demonstrated significant findings for preoperative values of NLR (AUC = 0.643, *p* < 0.001), yielding a sensitivity of 56.7% and specificity of 70.5%, with a cut-off value of 2.8; SII (AUC = 0.655, *P* < 0.001) yielded a sensitivity of 75.6% and specificity of 50.0%, with a cut-off value above 1066; SIRI (AUC = 0.664, *P* < 0.001) yielded a sensitivity of 63.3% and specificity of 67.5%, with a cut-off value above 1.05. All the data are compiled in Table 2, with Fig. 1 showing the ROC curve plots for NLR, SII, and SIRI.

**Table 2:** Comparison of AUC and cut-off values between the systemic inflammation biomarkers

	AUC	95% CI	*P*-value	Cut-off value	Sensitivity	Specificity
NLR	0.643	0.585–0.699	< 0.001	2.8	56.7%	70.5%
SII	0.655	0.597–0.709	< 0.001	1066	75.6%	50.0%
SIRI	0.664	0.607–0.718	< 0.001	1.05	63.3%	67.5%

*AUC* area under the curve, *CI* confidence interval, *NLR* neutrophil-to-lymphocyte ratio, *SII* systemic inflammatory index, *SIRI* systemic inflammation response index

### Univariable and multivariable analysis

We performed the univariate logistic regression analysis, in which preoperative statin use, type of surgery, duration of surgery, preoperative laboratory parameters including white blood cells (WBC) and fasting plasma glucose (FBG), and all the inflammatory indicators (NLR, SII, SIRI ) were marked as significant risk factors for prevalence of postoperative SIRS (Supplementary Table S2). Specifically, among the inflammatory indicators, an NLR level exceeding 2.8 (odds ratio [OR] = 3.25, 95% confidence interval [CI] 1.93–5.46, *p* < 0.001), an SII level exceeding 1066 (OR = 3.09, 95% CI 1.72–5.55, *p* < 0.001), and a SIRI level exceeding 1.05 (OR = 3.59, 95% CI 2.13–6.04, *p* < 0.001) were significantly associated with the occurrence of postoperative SIRS.

The significant parameters identified in the univariable analysis were subsequently validated through multivariable analysis. The multivariate analysis demonstrated that NLR ≥ 2.8, SII ≥ 1066, and SIRI ≥ 1.05 remained independent predictors of postoperative SIRS (Table 3). Additionally, the Hosmer-Lemeshow test indicated a good fit for all regression models, as evidenced by non-significant results (NLR ≥ 2.8, *P* = 0.683; SII ≥ 1066, *P* = 0.983; SIRI ≥ 1.05, *P* = 0.646).

**Table 3:** Univariate and multivariate analyses for predicting SIRS after cardiac surgery with cardiopulmonary bypass

Variables	Univariate analysis			Multivariate analysis		
	OR	95% CI	*P*-value	OR	95% CI	*P*-value
NLR ≥ 2.8	3.25	1.93–5.46	< 0.001	1.94	1.06–3.77	0.034
SII ≥ 1066	3.09	1.72–5.55	< 0.001	2.74	1.53–4.92	< 0.001
SIRI ≥ 1.05	3.59	2.13–6.04	< 0.001	2.46	1.30–4.67	0.006

Multivariable model was adjusted for preoperative statin use, type of surgery, duration of surgery, white blood cells and fasting plasma glucose

*OR* odds ratio, *CI* confidence interval, *NLR* neutrophil-to-lymphocyte ratio, *SII* systemic inflammatory index, *SIRI* systemic inflammation response index

### Comparison of postoperative outcomes between the SIRS and non-SIRS groups

Table 4 compares postoperative outcomes between the SIRS group and non-SIRS group. Patients with postoperative SIRS exhibited significantly longer median ICU stay [4 (2–6) vs. 2 (2–4) days, *P* < 0.001] and prolonged mechanical ventilation duration [17 (7–26) vs. 13 (6–18) hours, *P* < 0.001], alongside a higher rate of ICU readmission (10.0% vs. 3.7%, *P* = 0.034). The all-cause long-term mortality was markedly elevated in the SIRS group (27.8% vs. 16.0%, *P* = 0.020). No intergroup differences were observed in total hospital stay (*P* = 0.286) or in-hospital mortality (*P* = 0.366).

**Table 4:** Comparison of postoperative outcomes of the patients

	Non-SIRS ( *n* = 200)	SIRS ( *n* = 90)	*P*-value
ICU stay, median (IQR), days	2(2–4)	4(2–6)	< 0.001
Hospital stay, median (IQR), days	21(17–25)	21(18–27)	0.286
MV duration, median (IQR), hours	13(6–18)	17(7–26)	< 0.001
Readmission to the ICU, n (%)	7(3.7)	9(10.0)	0.034
In-hospital mortality, n (%)	2(1.0)	2(2.2)	0.366
All-cause long-term mortality, n (%)	32(16.0)	25(27.8)	0.020

*IQR* interquartile range, *MV* mechanical ventilation

### Subgroup analyses

Subgroup analyses were utilized to investigate the consistency of the predictive model for postoperative SIRS, defined by NLR ≥ 2.8, SII ≥ 1066, and SIRI ≥ 1.05, across different groups categorized by gender, age, BMI, diabetes, and hypertension (Table 5). Numerically different predictive effects were observed across subgroups. However, the P values for interaction in all subgroup comparisons were greater than 0.05, indicating that there was no statistically significant heterogeneity in the predictive value of these inflammatory indices for postoperative SIRS across different subgroups.

**Table 5:** Subgroup analyses for predicting SIRS after cardiac surgery with cardiopulmonary bypass

Subgroups	NLR ≥ 2.8	SII ≥ 1066	SIRI ≥ 1.05
	OR (95% CI)		
Gender			
Males (*n* = 130)	2.36 (0.85, 6.54)	2.10 (0.84, 5.13)	1.89 (1.13, 3.15)*
Females (*n* = 160)	1.50 (0.650, 3.47)	3.50 (1.59, 7.69) *	1.42 (0.92, 2.18)
* P* for interaction	0.444	0.324	0.393
Age subgroup			
65–69 (*n* = 104)	2.11 (0.64,6.95)	2.28 (0.84, 6.23)	4.20 (1.35, 13.11) *
70–74 (*n* = 123)	2.37 (0.86, 6.55)	4.81 (1.72, 13.48) *	3.06 (1.08, 8.63) *
≥ 75 (*n* = 63)	0.89 (0.17, 4.60)	1.62 (0.37, 7.11)	0.35 (0.06, 2.20)
P for interaction	0.459	0.414	0.241
BMI subgroup			
< 22 kg/m^2^ (*n* = 91)	1.28 (0.41, 4.03)	2.42 (0.89,6.52)	3.51 (1.12,11.06)*
22–27 kg/m^2^ (*n* = 150)	2.41 (0.94, 6.21)	3.36 (1.34,8.42) *	1.41 (0.54,3.69)
> 27 kg/m^2^ (*n* = 49)	1.67 (0.26, 10.86)	4.76 (0.78, 29.00)	4.86 (0.79, 29.98)
* P* for interaction	0.941	0.377	0.468
Hypertension			
Yes (*n* = 147)	1.58 (0.62, 4.00)	2.93 (1.29, 6.70) *	1.88 (0.78, 4.51)
No (*n* = 143)	2.29 (0.89, 5.88)	2.50 (1.06, 5.91) *	3.32 (1.23, 9.00) *
* P* for interaction	0.348	0.514	0.339
Diabetes			
Yes (*n* = 33)	1.40 (0.54, 36.35)	1.54 (0.82, 2.89)	4.23 (0.16, 113.12)
No (*n* = 257)	2.05 (1.05,4.01) *	2.12 (1.15, 3.90) *	2.49 (1.27, 4.88) *
* P* for interaction	0.581	0.056	0.403

*OR* odds ratio, *CI* confidence interval, *NLR* neutrophil-to-lymphocyte ratio, *SII* systemic inflammatory index, *SIRI* systemic inflammation response index, *BMI* body mass index

^*^P < 0.05

## Discussion

To our knowledge, this study is the first to investigate the potential effectiveness of preoperative inflammatory indexes in the prediction of SIRS for older patients following cardiac surgery with CPB. The multivariable regression analysis indicated that preoperative values of the NLR exceeding 2.8, SII greater than 1066, and SIRI above 1.05 are correlated with an increased incidence of postoperative SIRS. Furthermore, the analyses of subgroups and interactions corroborated the robustness of the associations among these variables. Besides, patients with postoperative SIRS had longer ICU stays, extended mechanical ventilation, higher ICU readmission rates, and increased long-term mortality.

Older patients undergoing cardiac surgery represent a uniquely vulnerable population, not merely due to chronological age but because of underlying biological shifts in immune homeostasis. Immunosenescence alters inflammatory reactions to surgical stress, creating a paradoxical phenotype: chronic low-grade inflammaging coexists with blunted acute inflammation and impaired immune surveillance [14]. Even without injury or infection, older patients sustain high baseline interleukin-6 (IL-6) and tumor necrosis factor-alpha (TNF-α) [7]. Such pre-inflammatory priming restricts immune flexibility during CPB, triggering either futile excessive inflammation or insufficient responses unable to limit tissue damage. Aging thus does not merely suppress immunity, but establishes a maladaptive equilibrium that renders patients prone to both hyperinflammatory SIRS and postoperative infectious complications.

Aligned with the age-specific susceptibility of the immune system, the traditional perspective that postoperative SIRS is simply a temporary and harmless postoperative occurrence has been contested by a growing body of clinical evidence. Recent research suggests that SIRS in the geriatric population is increasingly acknowledged as a precursor to severe complications [15–17]. Although emerging evidence challenges the assumption that advanced age uniformly increases SIRS risk, even mild SIRS can precipitate significant hemodynamic instability and arrhythmias in older patients, increasing the risk of prolonged mechanical ventilation, acute kidney injury, and cognitive decline [18]. In our study, the incidences of SIRS was 31.0% (90/290) within postoperative 3 days, consistent with findings from other studies [16, 19]. Patients with postoperative SIRS faced longer ICU stays, extended mechanical ventilation, higher ICU readmission rates, and increased long-term mortality, highlighting the need to predict SIRS before cardiac surgery.

The 2016 Sepsis-3 consensus updated criteria for infection-related organ dysfunction but retained the classic SIRS definition for sterile surgical inflammation. The 1992 ACCP/SCCM consensus defined SIRS as encompassing inflammatory responses from both infectious and sterile causes, including CPB and surgical trauma [20]. Our study aligns with this framework by excluding patients with preoperative infections and primarily focusing on non-infectious inflammatory responses after cardiac surgery. While Sepsis-3 uses ΔSOFA ≥ 2 to define sepsis, it lacks a standardized tool for measuring sterile post-cardiotomy inflammation, making SIRS the most commonly used metric in cardiothoracic surgical research. Furthermore, large cardiac surgical cohorts confirm that counting satisfied standard SIRS criteria reliably stratifies inflammatory severity and predicts postoperative complications [9, 21, 22], and consistent use of this universal definition facilitates direct comparison with prior perioperative inflammation research.

Despite the routine administration of preoperative antimicrobial prophylaxis in various specialized surgical procedures for older adult patients, the consistently high incidence of postoperative SIRS indicates that this approach may not effectively prevent its occurrence [2]. In addition, current management strategies are mostly reactive, starting only after SIRS appears, missing a key prevention window. This method is ineffective in changing outcomes, especially for older adults with reduced organ reserve and irregular immune responses, making them more prone to inflammation. Therefore, the preoperative prediction of the postoperative inflammatory response in individual cardiac surgery patients represents a crucial initial step towards developing a more targeted strategy for the prophylaxis of SIRS.

Recently, peripheral blood-derived composite inflammatory indices from routine blood tests have been widely applied in clinical practice, particularly for predicting postoperative SIRS, due to their cost-effectiveness and ready availability. A retrospective cohort study of 5595 older patients undergoing elective surgery found that a high preoperative CHR is significantly associated with a higher risk of postoperative SIRS [23]. According to Mehmet et al., the PLR before surgery is a cost-effective biomarker for predicting postoperative SIRS, while Tang et al. found that NLR were reliable indicators of SIRS after percutaneous nephrolithotomy (PCNL) [11, 12]. Subsequent large retrospective and prospective PCNL cohorts consistently supported the predictive utility of PLR and NLR [24, 25]. Moreover, in patients with intestinal obstruction, SII > 1792 was a risk factor for postoperative sepsis [26].

In terms of cardiac surgery, higher preoperative inflammatory markers levels are significantly associated with worse outcomes, including postoperative atrial fibrillation (POAF), acute kidney injury, and an increased risk of long-term death [27–29]. A retrospective analysis conducted at a single center involving 682 patients identified that a NLR greater than 3.5 and a monocyte-to-lymphocyte ratio (MLR) exceeding 0.2 serve as significant predictors of all-cause long-term mortality following off-pump coronary artery bypass grafting revascularization [30]. Moreover, the NLR, MLR, and PLR were identified as independent predictors of postoperative delirium in a retrospective observational analysis enrolling 2095 cardiac surgery patients, whose clinical data were extracted from the MIMIC database [31]. Similarly, the NLR has been shown to predict in-hospital mortality among patients with acute type A aortic dissection (TAAD) [32]. Notably, there have been limited reports concerning the relationship between preoperative inflammatory markers and postoperative SIRS in older patients receiving cardiac surgery with CPB. Our research revealed that individuals with SIRS exhibit elevated levels of NLR, SII, and SIRI compared to those without SIRS. Furthermore, multivariate analysis revealed that preoperative NLR ≥ 2.8, SII ≥ 1066, and SIRI ≥ 1.05 were each significantly linked to the occurrence of SIRS following cardiac surgery, whereas CPB duration and preoperative white blood cell count were not independent predictive factors. A study in patients who underwent PCNL identified that NLR ≥ 2.16, and SII ≥ 480.37 were independently associated with the development of post-PCNL SIRS [33]. The variability in the cutoff values of those markers may be due to the different sample sizes, type of surgery and population characteristics.

Numerous studies have identified prolonged CPB duration is associated with greater SIRS risk [6, 34]. However, no intergroup differences were found in our cohort for key intraoperative variables. This balance suggests that factors other than surgical trauma significantly contribute to SIRS onset. According to the conclusion of Litmathe et al.’s prospective cohort study, preoperative activation of endothelial cells, leukocytes and lymphocytes-rather than the length of extracorporeal circulation itself-is the primary determinant of post-CPB SIRS onset [15]. So, older patients with higher preoperative NLR, SII and SIRI that reflect more severe chronic inflammatory priming mount an exaggerated, uncontrolled inflammatory cascade when exposed to equivalent CPB stimulation. Our study found a difference in preoperative statin use between the groups. However, in the multiple regression analysis, statin use was not an independent risk factor for SIRS. This aligns with a large RCT from STICS, which showed that short-term high-dose rosuvastatin did not reduce postoperative inflammation [35]. Beyond preoperative medication-related confounders, early postoperative complications such as bleeding, cardiac tamponade, IABP and surgical reexploration may trigger additional pro-inflammatory cytokine release, increasing systemic inflammation after cardiac surgery [4, 36]. Meanwhile, variable use of postoperative medications like vasoactive agents, glucocorticoids, and dexmedetomidine can alter inflammation and introduce unmeasured bias impacting SIRS occurrence [37, 38]. Notably, vasoplegia and hyperdynamic syndrome after cardiac surgery often overlap with standard SIRS criteria, mainly due to persistent tachycardia and compensatory tachypnea from systemic vasodilation and increased cardiac output. This overlap complicates distinguishing between true inflammatory SIRS and hemodynamic disturbances. Future prospective studies are needed that collect more details regarding early postoperative complications and postoperative medications to conduct a more comprehensive analysis of the influencing factors of postoperative SIRS. Moreover, the wide array of intraoperative, postoperative and hemodynamic confounding factors described above helps explain the limited discriminative power and relatively low AUC values of the three preoperative inflammatory biomarkers.

Our study has important limitations in addition those already noted. First, its retrospective design introduces potential selection bias. Second, it was a single-center study with small number of individuals evaluated, limiting major extrapolations. Thirdly, even after adjusting for multiple confounding variables, there remains the potential for residual unmeasured factors. In addition, although this study first discovered a correlation between high preoperative inflammatory markers and postoperative SIRS in geriatric patients with cardiac surgery, its AUC and sensitivity were not outstanding enough. Ultimately, certain studies highlight the significance of continuously monitoring inflammatory indexes, which was overlooked in this study due to data constraints.

## Conclusions

According to our results, increased preoperative NLR, SII and SIRI level is an independent risk factor for postoperative SIRS among older patients after cardiac surgery. The results of our study may serve as a valuable and economically feasible tool for the preoperative identification of patients at increased risk.

## Supplementary Information

Supplementary Material 1.

## Data Availability

The datasets used and/or analyzed in the current study are available from the corresponding author on reasonable requests.

## Abbreviations

SIRS: Systemic inflammatory response syndrome
NLR: Inflammatory indices including neutrophil-to-lymphocyte ratio
SII: Systemic inflammatory index
SIRI: Systemic inflammation response index
OR: Odds ratio
CPB: Cardiopulmonary bypass
CONUT: Controlling Nutritional Status
SD: Standard deviation
ROC: Receiver operating characteristic
AUC: Area under the curve
CI: Confidence interval
BMI: Body mass index
NYHA: New York Heart Association
COPD: Chronic obstructive pulmonary disease
CABG: Coronary artery bypass grafting
DOS: Duration of surgery
ACC: Aortic cross clamp
WBC: White blood cells
PLT: Platelet count
Hb: Hemoglobin
TSB: Total serum bilirubin
SUA: Serum uric acid
Cr: Creatine
TG: Triglyceride
HDL-C: High-density lipoprotein cholesterol
LDL-C: Low-density lipoprotein cholesterol
FPG: Fasting plasma glucose
eGFR: Estimated glomerular filtration rate
