# Supplementary material for: Preoperative inflammatory indicators predict postoperative SIRS after cardiac surgery with cardiopulmonary bypass in older patients
Source: BMC Geriatr. 2026 Aug 7;26:1188. doi: 10.1186/s12877-026-08075-5 (PMC13595728; doi:10.1186/s12877-026-08075-5)
Supplement: Supplementary file 1 [file 12877_2026_8075_MOESM1_ESM.docx]

Appendix S1. Standard Structured Telephone Follow-up Questionnaire

## Opening Remarks

Hello,

My name is (	) and I’m calling from the Department of Cardiac Surgery. May I speak to (patient’s full name)?

If the patient is unavailable at present: Could you please tell me a suitable time to contact him/her again?

**Module 1: Vital status inquiry (For family proxies of deceased patients)**

We are deeply sorry for your loss. For hospital clinical quality control and research follow-up, could you provide the following information:

Date of death:

Primary cause of death:

Additional notes:

**Module 2: Interview consent & baseline confirmation (For living patients)**

If the patient answers the call directly:

This call is for postoperative follow-up of your cardiac surgery performed in our center. The whole interview will last 5 minutes. Is it convenient for you to participate in this survey now?

□ Yes, proceed with the questionnaire

□ No, reschedule the follow-up call

**Follow-up Outcome: Unplanned all-cause hospital readmission within the study follow-up window**

Has any unplanned hospital readmission occurred after your discharge from cardiac surgery?

□ No readmission during follow-up

□ 1 episode of unplanned readmission

□ ≥2 episodes of unplanned readmission

**Itemized recording for each readmission event**

Episode 1: Admission date	| Primary admission diagnosis

Episode 2: Admission date	| Primary admission diagnosis

Episode 3: Admission date	| Primary admission diagnosis

Supplementary notes for readmission:

**Module 3: End of interview & contact information archive**

Confirm current valid patient contact number:

Backup emergency family contact name & phone:

Additional follow-up remarks:

Quality Control Record (Mandatory)

Date of telephone follow-up: Interviewer signature:

Figure S1. Flow chart of patient selection and classification.

Table S1. Supplementary perioperative medication and intraoperative support characteristics.

| Variables | Non-SIRS ( n=200) | SIRS ( n=90) | *P*-value |
| --- | --- | --- | --- |
| Preoperative medications n (%) |  |  |  |
| ARBs | 11.0% | 15.6% | 0.276 |
| ARNI | 7.0% | 7.8% | 0.813 |
| ARAs | 62.5% | 61.1% | 0.822 |
| Beta blockers | 42.0% | 46.7% | 0.458 |
| Calcium channel blockers | 13.5% | 17.8% | 0.343 |
| Loop diuretic continuous infusion | 60.5% | 60.0% | 0.936 |
| Statins | 26.0% | 38.9% | 0.027 |
| Beraprost sodium | 12.5% | 10.0% | 0.540 |
| Steroids | 0.5% | 0.0% | 0.690 |
| Antiplatelet agents | 6.5% | 11.1% | 0.179 |
| Anticoagulants | 33.5% | 34.4% | 0.875 |
| Trimetazidine | 3.5% | 6.7% | 0.228 |
| Digoxin | 12.0% | 14.4% | 0.564 |
| Intraoperative medication n (%) |  |  |  |
| Norepinephrine | 97.0% | 97.8% | 0.708 |
| Phenylephrine | 4.5% | 3.3% | 0.644 |
| Vasopressin | 7.0% | 2.2% | 0.099 |
| Dopamine | 95.0% | 98.9% | 0.109 |
| Dobutamine | 13.0% | 17.8% | 0.285 |
| Milrinone | 95.5% | 97.8% | 0.348 |
| Levosimendan | 41.5% | 43.3% | 0.770 |
| Cardioplegia | 66.5% | 72.2% | 0.333 |
| Intraoperative MCS |  |  |  |
| IABP | 1.5% | 5.6% | 0.113 |
| ECMO | 1.5% | 2.2% | 0.647 |
| Intraoperative transfusion |  |  |  |
| RBC transfusion | 41.0% | 44.4% | 0.583 |
| Non-RBC blood products transfusion | 43.5% | 45.6% | 0.744 |

Note: ARBs, angiotensin II receptor blockers; ARNI, angiotensin receptor/neprilysin inhibitor; ARAs, aldosterone receptor antagonists; MCS, mechanical circulatory support; IABP, intra-aortic balloon counterpulsation; ECMO, extracorporeal membrane oxygenation; RBC, Red blood cell.

Table S2. Univariate analyses for predicting SIRS after cardiac surgery with cardiopulmonary bypass.

| Variables | Univariate analysis | | |
| --- | --- | --- | --- |
|  | OR | 95%CI | *P*-value |
| Age, years | 0.99 | 0.93-1.04 | 0.635 |
| Male sex | 1.02 | 0.62-1.69 | 0.930 |
| BM, kg/m^2^ | 0.98 | 0.92-1.05 | 0.597 |
| NYHA functional class ≥3 | 0.75 | 0.45-1.24 | 0.259 |
| Co-morbidities |  |  |  |
| COPD | 4.52 | 0.40-50.54 | 0.220 |
| Diabetes | 1.13 | 0.52-2.43 | 0.762 |
| Hypertension | 1.04 | 0.63-1.71 | 0.875 |
| Stroke | 0.85 | 0.34-2.12 | 0.732 |
| CONUT ≥ 2 | 1.37 | 0.81-2.32 | 0.236 |
| Preoperative medications n (%) |  |  |  |
| ARB | 1.49 | 0.72-3.07 | 0.279 |
| ARNI | 1.12 | 0.44-2.88 | 0.813 |
| ARAs | 0.94 | 0.57-1.57 | 0.822 |
| Beta blocker | 1.21 | 0.73-1.99 | 0.459 |
| Calcium channel blockers | 1.39 | 0.71-2.72 | 0.344 |
| Loop diuretic continuous infusion | 0.98 | 0.59-1.63 | 0.936 |
| Statins | 1.81 | 1.07-3.07 | 0.028 |
| Beraprost sodium | 0.78 | 0.35-1.74 | 0.541 |
| Antiplatelet agents | 1.80 | 0.76-4.27 | 0.184 |
| Anticoagulants | 1.04 | 0.62-1.76 | 0.875 |
| Trimetazidine | 1.97 | 0.64-6.04 | 0.236 |
| Digoxin | 1.24 | 0.60-2.56 | 0.564 |
| Type of surgery |  |  |  |
| Valve | Reference |  |  |
| Valve + CABG | 2.00 | 1.00-4.01 | 0.051 |
| Aorta | 5.50 | 1.91-15.83 | 0.002 |
| Other | 1.34 | 0.72-2.51 | 0.360 |
| Intraoperative characteristics |  |  |  |
| Intraoperative procedural durations |  |  |  |
| DOS (min) | 1.00 | 1.00-1.01 | 0.013 |
| CPB time (min) | 1.00 | 1.00-1.01 | 0.120 |
| ACC time (min) | 1.00 | 1.00-1.01 | 0.224 |
| Intraoperative medication n (%) |  |  |  |
| Norepinephrine | 1.36 | 0.27-6.88 | 0.709 |
| Phenylephrine | 0.73 | 0.19-2.77 | 0.646 |
| Vasopressin | 0.30 | 0.07-1.36 | 0.118 |
| Dopamine | 4.68 | 0.59-37.16 | 0.144 |
| Dobutamine | 1.45 | 0.73-2.86 | 0.287 |
| Milrinone | 2.07 | 0.44-9.80 | 0.357 |
| Levosimendan | 1.08 | 0.65-1.78 | 0.770 |
| Cardioplegia | 1.31 | 0.76-2.26 | 0.333 |
| Intraoperative MCS |  |  |  |
| IABP | 3.86 | 0.90-16.53 | 0.07 |
| ECMO | 1.49 | 0.25-9.09 | 0.664 |
| Intraoperative transfusion |  |  |  |
| Red blood cell transfusion | 1.15 | 0.70-1.90 | 0.583 |
| Non-RBC blood products transfusion | 1.09 | 0.66-1.79 | 0.744 |
| Laboratory parameters |  |  |  |
| WBC, ×10 ^9^/L | 1.18 | 1.08-1.28 | <0.001 |
| Hb, g/L | 1.00 | 0.98-1.01 | 0.642 |
| TSB, mg/dL | 1.43 | 0.88-2.32 | 0.145 |
| SUA, umol/L | 1.00 | 1.00-1.00 | 0.625 |
| Cr ,mg/dL | 1.00 | 0.99-1.01 | 0.703 |
| TG, mmol/L | 1.14 | 0.87-1.50 | 0.343 |
| HDL-C, mmol/L | 0.88 | 0.44-1.74 | 0.711 |
| LDL-C, mmol/L | 0.90 | 0.66-1.24 | 0.518 |
| Albumin, g/L | 1.00 | 0.91-1.10 | 0.975 |
| FPG, mmol/L | 1.19 | 1.02-1.39 | 0.026 |
| eGFR, mL/min/1.73 m^2^ | 1.00 | 0.99-1.01 | 0.984 |
| NLR ≥ 2.8 | 3.25 | 1.93-5.46 | <0.001 |
| SII ≥ 1066 | 3.09 | 1.72-5.55 | <0.001 |
| SIRI ≥1.05 | 3.59 | 2.13-6.04 | <0.001 |

Note: BMI, body mass index; NYHA, New York Heart Association; COPD, chronic obstructive pulmonary disease; CONUT, Controlling Nutritional Status; CABG, coronary artery bypass grafting; DOS, duration of surgery; CPB, cardiopulmonary bypass; ACC, aortic cross clamp; WBC, white blood cells; Hb, hemoglobin; TSB, total serum bilirubin; SUA, serum uric acid; Cr, creatine; TG, triglyceride; HDL-C, high-density lipoprotein cholesterol; LDL-C, low-density lipoprotein cholesterol; FPG, Fasting plasma glucose; eGFR, estimated glomerular filtration rate; NLR, neutrophil-to-lymphocyte ratio; SII, systemic inflammatory index; SIRI, systemic inflammation response index.
